# Empagliflozin suppresses hedgehog pathway, alleviates ER stress, and ameliorates hepatic fibrosis in rats

**DOI:** 10.1038/s41598-023-46288-5

**Published:** 2023-11-03

**Authors:** Nourihan Abdalla, Dina M. Abo-ElMatty, Sami Saleh, Maivel H. Ghattas, Nesreen Nabil Omar

**Affiliations:** 1 Department of Biochemistry, Faculty of Pharmacy, Modern University for Technology and Information, Mokattam, Cairo, 11585 Egypt; 2 Department of Biochemistry, Faculty of Pharmacy, Suez Canal University, Ismailia, 41522 Egypt; 3 Department of Medical Biochemistry, Faculty of Medicine, Port Said University, Port Said, Egypt

**Keywords:** Biochemistry, Liver fibrosis

## Abstract

Worldwide mortality from hepatic fibrosis remains high, due to hepatocellular carcinoma and end stage liver failure. The progressive nature of hepatic fibrosis from inflammation to cicatrized tissues warrants subtle intervention with pharmacological agents that hold potential. Empagliflozin (Empa), a novel hypoglycemic drug with antioxidant and anti-inflammatory properties, has lately been proposed to have additional antifibrotic activities. In the current study, we examined the antifibrotic effect of the Empa through modulating the activity of hepatic stellate cells by hedgehog (Hh) pathway. We also assessed the markers of inflammatory response and endoplasmic reticulum (ER) stress. Male Albino rats were treated with either CCl_4_ (0.4 mg/kg twice/week) and/or Empa (10 mg/kg/day) for eight weeks. In this study, CCl_4_ rats had active Hh signaling as indicated by overexpression of Patched 1, Smoothened and Glioblastoma-2. CCl_4_ induced ER stress as CHOP expression was upregulated and ERAD was downregulated. CCl_4_-induced inflammatory response was demonstrated through increased levels of TNF-α, IL-6 and mRNA levels of IL-17 while undetectable expression of IL-10. Conversely, Empa elicited immunosuppression, suppressed the expression of Hh markers, and reversed markers of ER stress. In conclusion, Empa suppressed CCl_4_-induced Hh signaling and proinflammatory response, meanwhile embraced ER stress in the hepatic tissues, altogether provided hepatoprotection.

## Introduction

More than 20% of individuals subjected to chronic hepatic injury end up in developing fibrosis, which can progress to hepatocellular carcinoma (HCC) and or liver failure if left untreated^1^. As the liver lies at the interface between the heterogenous external environment and the rest of the body, daily injuries are inevitable. In the current study, CCl_4_ was deployed to induce liver fibrosis, an analog for a persistent insult and a well-documented standard model of fibrosis^2^.

Fibrosis can usually be reversed unless it is greatly expanded, so there comes a point when it becomes irreversible, which shortens survival to 2–4 years^3^. The constant chronic exposure of the hepatic tissues to insults provides a floor of damage that exceeds the regenerative response. Meanwhile, the regeneration reaction is a cascade of wound healing that, if left unchecked, scarring or fibrosis will take place^4^. Fibrosis is best described as inadequacy of replenishing damaged hepatocytes at a time when the hepatic stellate cells (HSCs) produce excess of extracellular matrix (ECM) to fill the voids in hepatic parenchyma^5^. Therefore, the liver tissues are viewed as repaired when they are dysfunctional.

The fixation of injured liver tissues ideally requires increased hepatocytes cellularity rather than augmented HSCs activity. HSCs are non-parenchymal hepatic cells resident at the bank of hepatic vessels and serve as a repository for vitamin A^6^. HSCs are considered flexible cells which can be triggered upon injury and acquire a phenotype called myofibroblast that secrets ECM^6^.

Disturbed homeostasis in wounded hepatic tissues depicts post-signaling of an unprecedented pathway. Hedgehog (Hh) pathway signals in infancy to enhance growth and maturation of rudimentary tissues, then it halts after tissue maturation^7^. However, damage- associated molecules act as ligands for Hh pathway, which is considered a morphogen that dictates the activation of its nearby cells, while remote cells remain dormant and prone to damage^1^. Given that Hh proteins are located on the primary cilia of HSCs^8^, the adaptive transition of latent HSCs into dynamic myofibroblast through Hh pathway is justified. When damage-associated molecules bind to the Hh receptor on primary cilia, Patched receptor 1 (Ptch1), the signal is transduced through activation of Smoothened protein (Smo) to activate the transcription factor, Glioblastoma-2 (Gli-2), which in turn transcribes proliferation genes^9^.

Damage- associated molecules are signals for danger since they are the products of dying cells, therefore in addition to enabling Hh signaling, they induce heavy trafficking of immune cells to eliminate cellular debris which has no way out as the liver is lumenless^10^. Although inflammation is part of the immune response to clear damage, repeated insults result in an exuberant inflammatory reaction that does more damage, leaving the case open^11^.

As the hepatic cells are overwhelmed with synthetic, metabolic, storage and detoxification functions, the cytosol of hepatocytes is piled with endoplasmic reticulum (ER) machinery to carry out these duties. Under stress, ER is highly susceptible to dysfunction, so it is armed with sensor proteins called the unfolded protein response (UPR)^12^. UPR detects and responds to damage by halting protein synthesis or directing protein to degradation by boosting ER-associated protein degradation (ERAD)^13^. If the stress is prolonged, the UPR is disengaged and activation of (CCAAT/enhancer binding protein) homologous protein (CHOP) takes place which activates apoptotic cell death pathways^14^.

Standard therapy is a pharmacological agent used in remediation of at-risk population, with the reward of an antifibrotic effect. In this sense, antidiabetics used to improve hyperglycemia in patients suffering from insulin resistance and diabetes, will benefit from the additional antifibrosis, which they desperately need^15^. Empagliflozin (Empa) is a sodium-glucose secondary transporter 2 (SGLT2) inhibitor that functions as an antidiabetic mediator and privileged with antioxidant and anti-inflammatory properties^16^. Empa has been shown to reduce steatosis and fibrosis in nonalcoholic fatty liver disease (NAFLD) patients with and without diabetes^17, 18^. Multiplicity of the studies assessed the metabolic effect of Empa in diet-induced hepatic fibrosis^19, 20^, however, the potential application of Empa in toxicity-induced hepatic fibrosis has not been explored. Insightfully, the anti-inflammatory action of Empa provides a mechanistic control of fibrosis^21^. Also, the implementation of Hh in the therapeutic action of Empa on CCl_4_-induced hepatic fibrosis is not clear. Lately, empagliflozin intervened with the hedgehog pathway to modulate carcinogenesis^22^. The aim of the current study is to estimate the potential ameliorative effects of Empa in in CCl_4_-induced hepatic fibrosis and unravel the impact of Empa as antioxidant and anti-inflammatory mediator in regulating Hh signaling, thereby affecting the entire process of liver fibrosis.

## Materials and methods

### Chemicals

Empagliflozin (Jardiance^®^) was obtained from Boehringer Ingelheim International GmbH, Ingelheim, Germany. Carbon tetrachloride (CCl_4_) was purchased from El-Nasr Chemical Co (Egypt).

### Animals and experimental design

Experiments were conducted on 32 5-week-old rats (Albino), 100–150 g housed in cages (8/cage) under controlled conditions. Animals were provided from the animal house of the National Organization for Drug Control and Research (NODCAR), Giza, Egypt. Animals were fed ad libitum and acclimated for two weeks prior to the experiment. The animals are housed at 22 ± 2 °C in light/dark cycles. All animal procedures and care were carried out according to the general guidelines of Scientific Research Ethics Committee of the Faculty of Pharmacy, Suez Canal University, Ismailia, Egypt. The experimental protocol was reviewed and approved by the Scientific Research Ethics Committee- Faculty of Pharmacy- Suez Canal University with the approval no. 20212 MA5. This is in compliance with the ARRIVE guidelines and was carried out in accordance with the U.K. Animals (Scientific Procedures) Act, 1986 and associated guidelines, EU Directive 2010/63/EU for animal experiments. Rats were divided equally into four groups and all the drugs were administrated via gastric lavage. The first group feeds on basal diet and received corn oil (0.25 ml, i.p) twice/week as vehicle for CCl_4_, and normal saline daily (10 ml/kg/day, orally) as vehicle for empagliflozin for 8 consecutive weeks; the second group are rats treated with empagliflozin daily (10 mg/kg/day in 10 ml/kg/day normal saline via gastric lavage) and corn oil (0.25 ml, i.p) twice/week for 8 consecutive weeks; the third group are rats with induced liver fibrosis by injecting CCl_4_ twice/week (0.4 mg/kg CCl_4_ in 0.25 ml/kg corn oil via intraperitoneal injection)^23^ for 8 consecutive weeks and the fourth group are rats treated with CCl_4_ twice/week (0.4 mg/kg CCl_4_ in 0.25 ml/kg corn oil via intraperitoneal injection) concurrently with empagliflozin daily (10 mg/kg/day in 10 ml/kg/day normal saline via gastric lavage)^24^ or 8 consecutive weeks. At the end of treatment, rats were decapitated, blood was collected, and hepatic tissues were isolated.

### Blood collection and tissue processing

After blood was drawn from the retro-orbital plexus of all animals, rats of different groups were sacrificed by rapid decapitation. Blood was allowed to clot, and serum was obtained by centrifugation at 3000×*g* for 15 min. After decapitation, hepatic tissues were excised and immersed in a cold 0.9% NaCl solution. The weight of the liver was recorded to calculate the liver index. Samples from each liver were used for histopathology and transmission electron microscopy studies (specimens in formaldehyde and glutaraldehyde, respectively). The rest of the hepatic tissues were immediately frozen on dry ice and stored at − 80 °C. livers were homogenized in 0.1 mol/l Tris–HCl buffer (pH 7.4). Aliquots of the homogenate were used to assess markers of oxidative stress, the immune response, the Hh pathway fibrosis and the ER stress.

### Real time PCR (RT-PCR)

RNeasy Plus Mini kits (Qiagen) was used for the extraction of RNA from the liver tissues. RNA was eluted in a volume of 60 µL. NanoDrop 2000 (Thermo Scientific, USA) was utilized to assess RNA yield and purity which was then stored at − 80 °C. QuantiTect Reverse Transcription Kit (Qiagen) (cat. no. 205310) and RT Primer Mix (1 µg) were used to reverse-transcribe RNA into first strand cDNA. QuantiTect SYBR Green PCR Kit (cat. no. 204141) and Applied Biosystems StepOnePlus instrument were employed to examine gene expression. HotStarTaq DNA polymerase activation required an initial PCR activation step at 95 °C for 15 min. Standard fast thermal cycling parameters of 40 cycles at 95 °C for 15 s and 60 °C for 60 s were used as recommended by the manufacturer. All reactions were repeated three times. The internal control was beta actin. The chosen primer sequences are shown in Table 1. C_t_ values were applied to quantify the results. The definition of C_t_ is the PCR cycle threshold corresponding to the first detection of the amplification product and expressed as the ratio of target to control.

**Table 1:** Genes list used for RQ-RT-PCR validation.

Gene	Forward primer	Reverse primer	Accession number	Amplicon size
IL-17	AACGCCGAGGCCAATAACTT	GGTAGGCGTGATGCACCAAA	NC_051344.1	203
IL-10	TTCAAATGGCACGCGTGATG	CAGCACCTCCCCTCCCTAAT	NC_051348.1	181
CHOP	TCTTGAGTCTAATACGTCGATCAT	CGGTTTCTGCTTTCAGGTGT	NM_001109986.1	144
ERAD	AATCTGCTCTGCCGAGTGTC	AAGGGCAAGTGTAAGGTCGG	NC_051341.1	195
α-SMA	GGGAGCATCATCACCAGCAA	TGGTTGGGAGTGACCGTAGG	NC_051336.1	180
Ptch-1	CTACACTCAAGCAGGCGAGG	GTGCAAATGGAATCCCAGGC	NC_051352.1	212
Smo	ACTCCAGGTATGAGGGCTCTG	GGCTTGTTCTTCTGGTGGCA	NC_051339.1	260
Gli-2	CTGCACGCAAAGAGGTGATT	GGCCACTTTTGTCTGCGTTT	NC_051348.1	145
β-actin	AAGGCTATAGTCACCTCGGG	GGTAATAATGCGGCCGGTCT	NC_000071.7	144

### Evaluation of hepatotoxicity index

Levels of AST and ALT in serum were determined colourimetrically according to the method of Reitman and Frankel^25^, using available commercial kits (Biodiagnostics, Giza, Egypt). Liver index was calculated according to the formula: (liver weight/body weight) × 100.

### Evaluation of oxidative stress markers

Superoxide dismutase (SOD) as well as reduced glutathione (GSH) levels in the hepatic tissues were determined colourimetrically using commercial kits purchased from Bio-diagnostics, Giza, Egypt, in alignment with the standard procedures. Malondialdehyde (MDA) was used as a surrogate for lipid peroxidation. It was determined according to the method of Uchiyama and Mihara^26^. 1,1,3,3-tetraethoxypropane was used as a standard and results were expressed as nmol of MDA/g tissue using.

### Estimation of the activity of the immune system in hepatic tissues

Protein content in the liver tissues was determined colourimetrically using a commercially available kit (Spectrum diagnostics, Cairo, Egypt). Tissue TGF-β1 and IL-6 levels were assessed using ELISA kits obtained from Thermo Fisher Scientific, Inc., Germany (Catalog # BMS623-3 and BMS625 respectively), tissue TNF-α was measured using ELISA kits purchased from RayBiotech, Inc., USA (Catalog # ELR-TNFα-1), according to the manufacturer’s protocol. They were all measured relative to the estimated protein content. IL-17 and IL-10 levels were detected by Real Time PCR to identify the activation or suppression of the immune response.

### Markers of Hh pathway and ER stress

Hh pathway was tracked through assessment of the gene expression of Ptch1, Smo and Gli-2. The gene expressions of CHOP and ERAD were determined as representatives of ER stress.

### Markers of hepatic fibrosis

α-SMA levels were detected by Real Time PCR. Additionally, Masson’s trichrome staining was used to detect hepatic collagen content by picturing the blue color of collagen fibers.

### Histopathological examination

10% buffered formalin was used as a fixative for liver tissue samples for a whole night. Afterwards, they were washed then dehydrated using series of graded ethanol (30–100%). Hepatic tissues were then embedded in blocks of paraffin. A rotatory microtome was used for slicing of 5 µm thick. Deparaffinization was done in xylene, then sections were gone through alcohol of descending concentration and stained with hematoxylin. 70% alcohol was used soaking, then staining was done with eosin. Alcohol was used as a cleanser and xylene was used to clear the sections. Eventually, the sections were fixed with Dibutyl phthalate Polystyrene Xylene DPX. A light microscope was applied to investigate the slides and report the histological variations^27^. For examination of collagen fibers, the 5 µm thick slices were stained with Masson's trichrome stain. Liver fibrosis was classified according to the Ishak fibrosis score by experienced pathologists. The Ishak score classifies liver fibrosis in seven stages (0–7). Stage 0 means no fibrosis, Stages 1: portal fibrosis of single portal fields, Stage 2: portal fibrosis > 50% of portal fields, Stage 3: portal fibrosis > 50% of portal fields with portal-to-portal bridging, Stage 4: portal fibrosis > 50% of portal fields portal-to-central bridging with occasional nodules, Stage 5: incomplete cirrhosis, Stage 6: complete cirrhosis^28^.

### Transmission electron microscopy (TEM)

Liver specimens were divided into small pieces (1 mm^3^), mixed with 2.5% glutaraldehyde 0.1 N PBS at room temperature for one hour. Osmium tetraoxide was applied for fixation for one more hour. Hepatic tissues were hydrated twice in 70, 90 and 100% acetone each, and in a 1:1 (acetone: resin) for few minutes. Hepatic tissue specimens were then embedded in epoxy resin. Ultrathin Sections (90 nm) were viewed via a transmission electron microscope, Jeol Jem- 1400, USA Inc.

### Data analysis

The average threshold cycle (C_T_) and the comparative ΔΔC_T_ method were calculated for the expression of the gene and normalized to the mean C_T_-value of β-actin. Fold change (RQ) was used to compare gene expression between samples, and it was calculated as 2^−ΔΔCT^^29^. The rest of the data are represented as (Mean ± SD). Statistical analyses were performed by applying one-way ANOVA followed by Tukey–Kramer as a post-hoc test. Probability level equal to 0.05 is considered significant. All statistical analyses were performed by Instat version 3 software package. Graph Pad Prism (ISI^®^ software, USA) version 8.4.3 software was employed for drawing graphs.

## Results

### Empa reverses CCl_4_-induced hepatocytes damage

**Figure 1:**
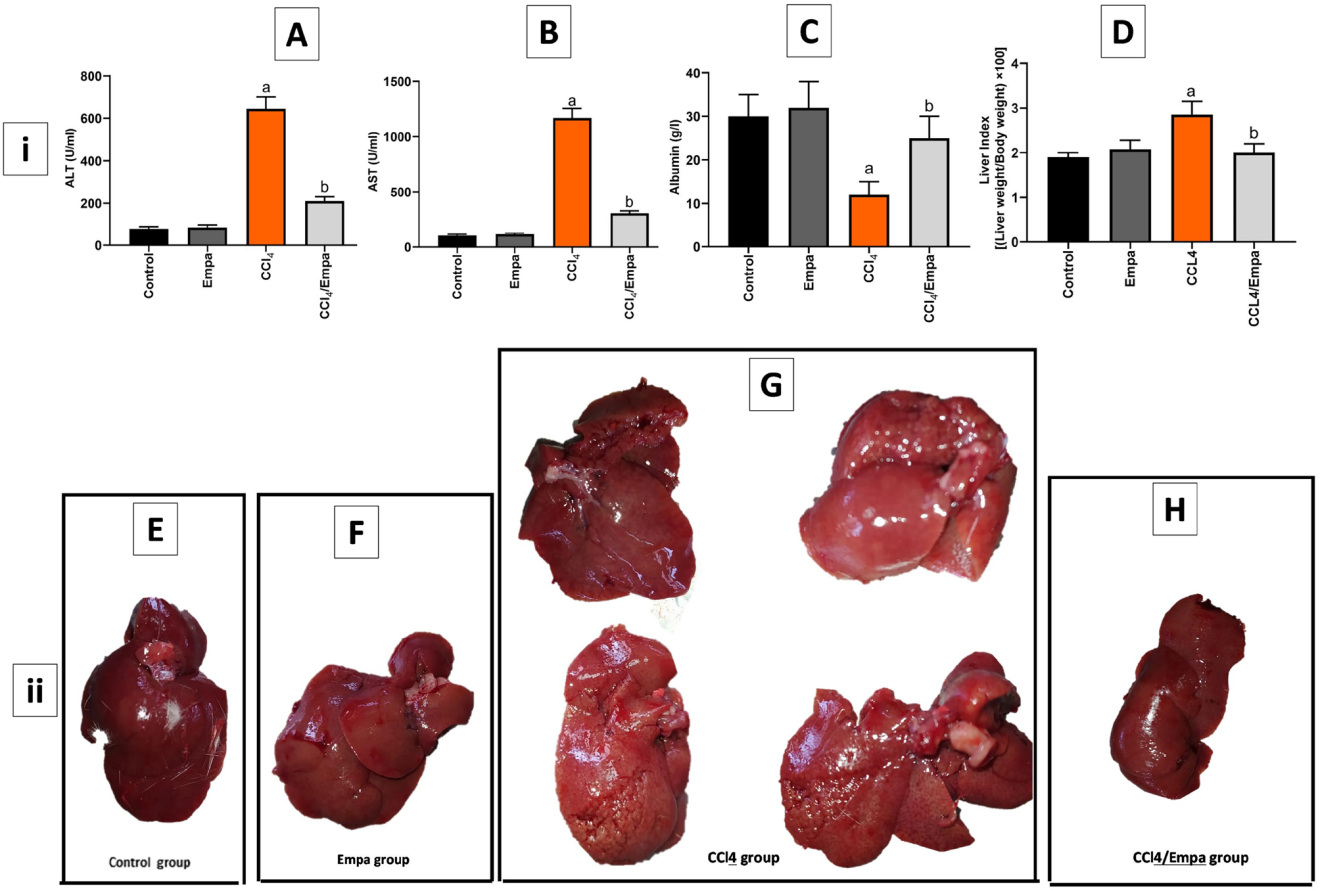
The effects of CCl4 and administration Empagliflozin on (i): Serum levels of ALT, AST, and albumin and (ii): Liver gross appearance. (**A**) ALT serum levels (U/ml) in different groups. (**B**) AST serum levels (U/ML) in different groups. (**C**) Albumin serum levels (g/l) in different groups. (**D**) Liver index. It is calculated according to the formula: (liver weight/body weight) × 100. (**E**) Liver gross appearance in control groups. (**F**): Liver gross appearance in Empa only groups. (**G**) Different photos of liver gross appearance in CCl4 groups. (**H**) Liver gross appearance in CCl4/Empa groups. Data are presented as the means ± SD. *P* < 0.05 is considered statistically significant; a, significant (*P* < 0.05) versus control; b, significant (*P* < 0.05) versus CCl4 group; one-way ANOVA followed by Bonferroni-corrected post hoc tests were conducted. Empa, Empagliflozin; ALT, Alanine transaminase; AST, Aspartate transaminase.

In the current study, CCl_4_ provoked a significant increase in serum ALT and AST levels by 8.2-fold and 11.4-fold respectively compared to the control group (*P* < 0.05). When CCl_4_-intoxicated rats were cotreated with Empa, serum ALT and AST levels were elevated only by 2.7-fold and threefold, respectively. Similarly, CCl_4_-intoxicated rats had significantly lower albumin levels with reference to the control group (2.5-fold) while rats concurrently administered with Empa had significantly higher albumin levels with regard to the CCl_4_ group (twofold) (Fig. 1). These deteriorating effects of CCl_4_ on liver cells integrity and functions were also apparent in hepatic architecture in Fig. 1ii where the gross appearance of the liver in the CCl_4_ group is mostly cicatrized, irregular, and rough and the tissues are hardened. On the other hand, the liver surface in the control and Empa groups was intact, regular, and smooth. When the CCl_4_ group was cotreated with Empa, the liver surface was regular, mostly smooth, and less stiff. As for the liver index, it was significantly increased in response to CCl_4_ administration relative to the control group (*P* < 0.05). When Empa was used in conjunction with CCl_4_, liver index was significantly decreased (*P* < 0.05) (Fig. 1D).

### Empa extinguished CCl_4_-induced oxidative stress

**Figure 2:**
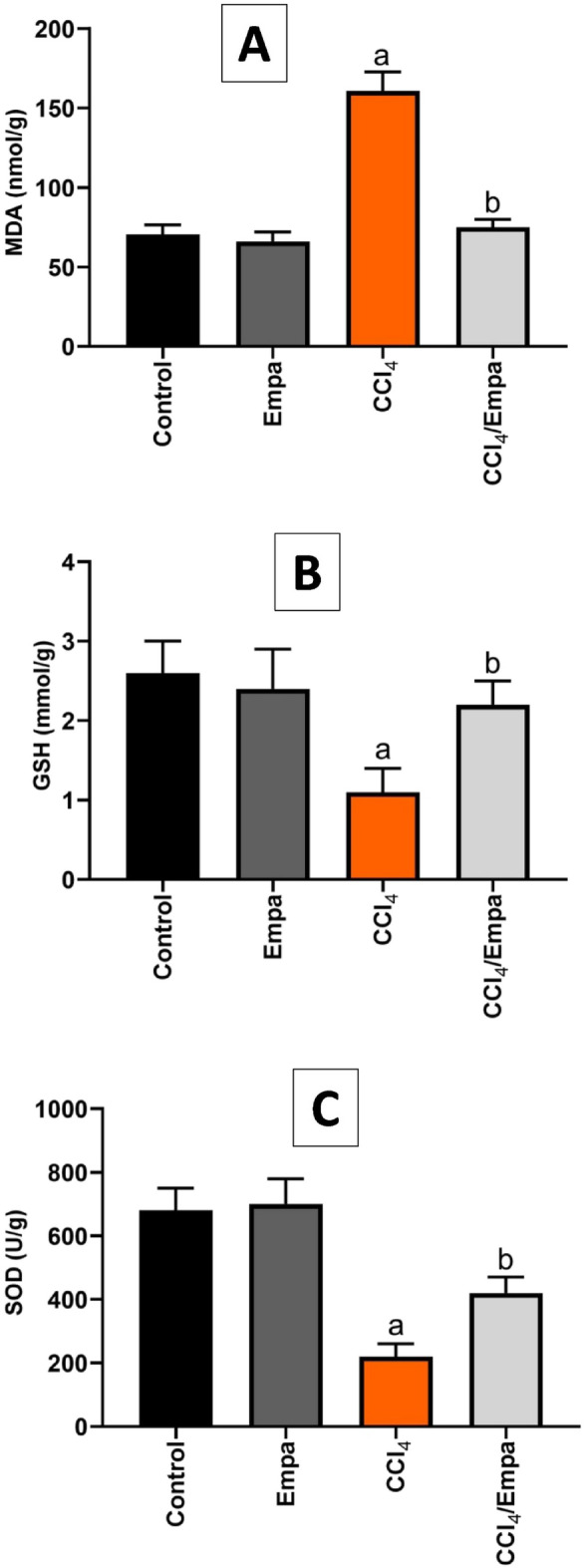
The effects of CCl_4_ and administration Empagliflozin on hepatic levels of oxidative stress markers. (**A**) MDA hepatic tissue levels (nmol/g) in different groups. (**B**) GSH hepatic tissue levels (mmol/g) in different groups. (**C**) SOD hepatic tissue levels (U/g) in different groups**.** Data are presented as the means ± SD. *P* < 0.05 is considered statistically significant; a, significant (*P* < 0.05) versus control; b, significant (*P* < 0.05) versus CCl_4_ group; one-way ANOVA followed by Bonferroni-corrected post hoc tests were conducted. Empa, Empagliflozin; MDA, Malondialdehyde; GSH, Reduced glutathione; SOD, Superoxide dismutase.

Chronic CCl_4_ administration prompted a significant 2.2-fold rise in MDA levels in the hepatic tissues compared to the control group. Meanwhile, a significant depletion of GSH by 2.3-fold and a remarkable decline in SOD activity by threefold after CCl_4_ administration were exhibited. After cotreatment with Empa, MDA levels returned to normal levels (2.1-fold decrease), GSH was replenished (2.2-fold increase) and SOD activity was preserved (1.9-fold increase) (Fig. 2).

### Empa modulates CCl_4_-elicited immune response

**Figure 3:**
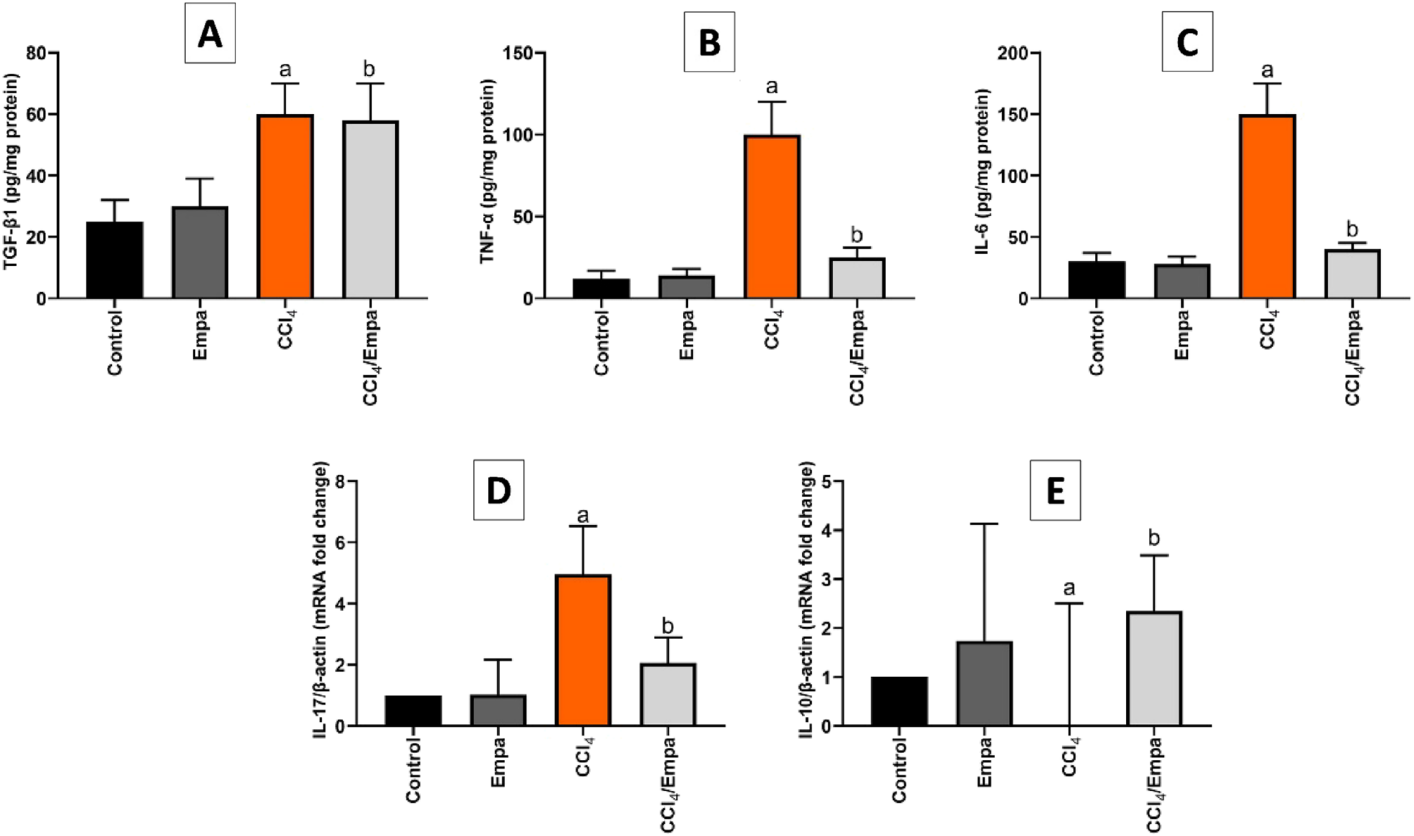
The effects of CCl_4_ and administration Empagliflozin on hepatic levels of inflammatory markers. (**A**) TGF-β1 hepatic tissue levels (pg/mg protein) in different groups. (**B**) TNF-α hepatic tissue levels (pg/mg protein) in different groups. (**C**) IL-6 hepatic tissue levels (pg/mg protein) in different groups. (**D**) IL-17 mRNA fold change relative to β-actin in different groups. (**E**) IL-10 mRNA fold changes relative to β-actin in different groups**.** Data are presented as the means ± SD. *P* < 0.05 is considered statistically significant; a, significant (*P* < 0.05) versus control; b, significant (*P* < 0.05) versus CCl_4_ group; one-way ANOVA followed by Bonferroni-corrected post hoc tests were conducted. Empa, Empagliflozin; TGF-β1, Transforming growth factor-beta1; TNF-α, Tumor necrosis factor alpha; IL-6, Interleukin-6; IL-17, Interleukin-17; IL-10, Interleukin-10.

CCl_4_-treated rats displayed active immune response, as the levels of inflammatory cytokines such as TNF-α, IL-6 and mRNA levels of IL-17 were significantly elevated by 7.1-fold, 5.3-fold, and 4.9-fold, respectively. While it was not feasible to detect the mRNA levels of IL-10, an anti-inflammatory cytokine. Empa elicited immunosuppression as distinguished by a fourfold and 3.75-fold reduction in TNF-α and IL-6 levels and a 2.45-fold decrease in of IL-17 expression. On the other hand, rats cotreated with Empa exhibited an active expression of IL-10 (2.3 times higher than the control group). As for TGF-β1, it was 2.4-fold upregulated in CCl_4_-treated rats. Empa/ CCl_4_ treated rats demonstrate no significant difference in TGF-β1 levels, an indication of immunomodulation (Fig. 3).

### Impact of Empa on hepatocyte and myofibroblasts

In this study, CCl_4_ enhanced the signaling of Hh pathways as Ptch-1, Smo and Gli-2 were overly expressed in hepatic tissues (11.5-fold, 8.2-fold and 3.8-fold increases). Concomitant Empa treatment in the CCl_4_ group resulted in a sixfold, 6.3-fold and 3.5-fold downregulation of the expression of Ptch-1, Smo and Gli-2, respectively.

**Figure 4:**
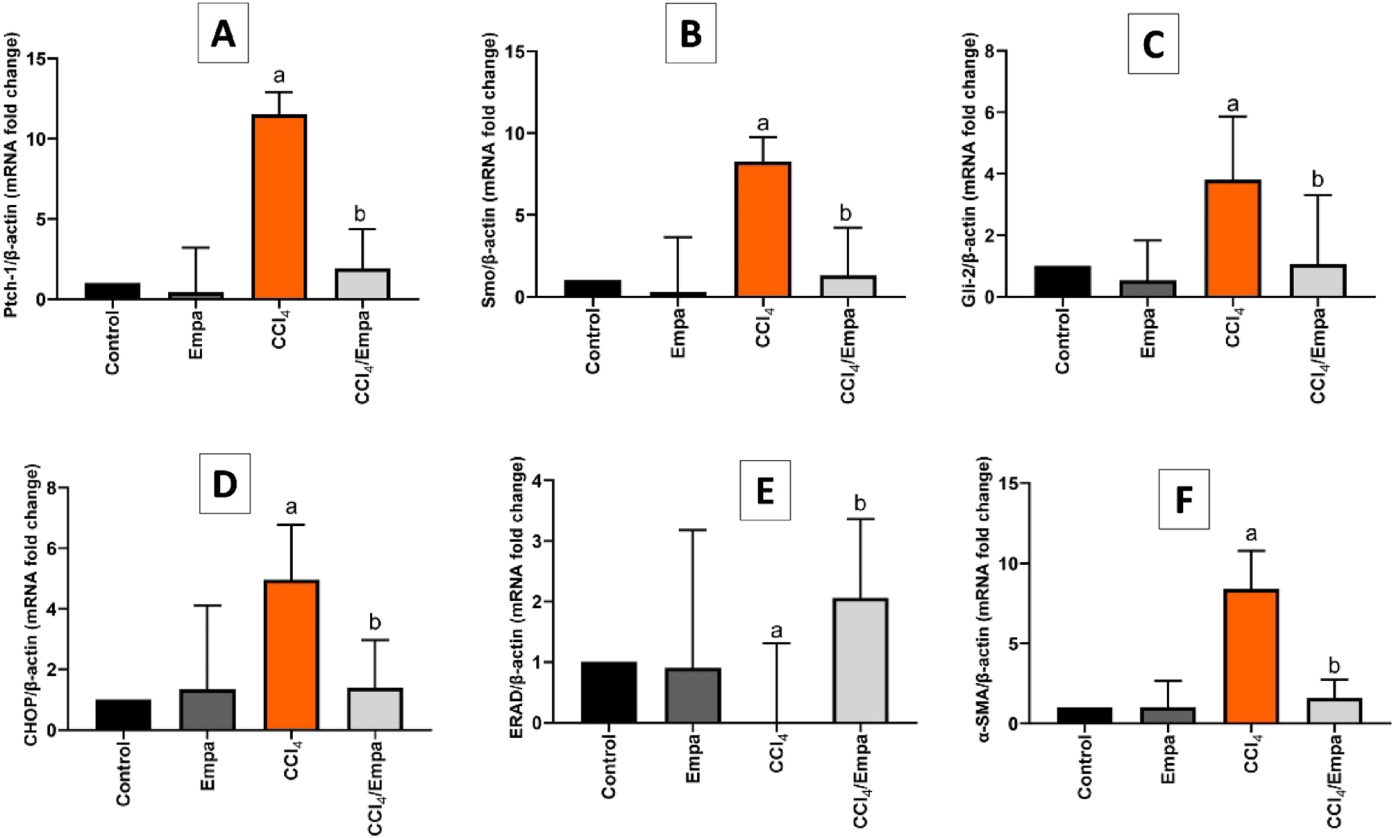
The effects of CCl_4_ and administration Empagliflozin on markers of hedgehog pathway, ER stress and fibrogenesis in the hepatic tissues. (**A**) Ptch-1 mRNA fold changes relative to β-actin in different groups. (**B**) Smo mRNA fold changes relative to β-actin in different groups. (**C**) Gli-2 mRNA fold change relative to β-actin in different groups. (**D**) CHOP mRNA fold changes relative to β-actin in different groups. (**E**) ERAD mRNA fold changes relative to β-actin in different groups. (**F**) α-SMA mRNA fold changes relative to β-actin in different groups**.** Data are presented as the means ± SD. *P* < 0.05 is considered statistically significant; a, significant (*P* < 0.05) versus control; b, significant (*P* < 0.05) versus CCl_4_ group; one-way ANOVA followed by Bonferroni-corrected post hoc tests were conducted. Empa, Empagliflozin; Ptch1, Patched 1; Smo, Smoothened; Gli-2, Glioblastoma-2; CHOP, The C/EBP homologous protein; ERAD, Endoplasmic reticulum-associated degradation; α-SMA, α-Smooth muscle actin.

Since ER occupies a large part of hepatocytes and enacts most of the tactile functions, it is considered as a deputy for operating cells^30^. ER responds to stress through an intricate pathway, for which CHOP is the endpoint for the failed defense^14^, while ERAD can armor cells against damage^13^. In this study, CCl_4_-induced hepatocytes injury was manifested through a 4.9-fold overexpression of CHOP and an abolished expression of ERAD. Empa cotreated group had diminished expression of CHOP by 3.7-fold and intensified expression of ERAD by 200-fold. Similarly, CCl_4_-induced fibrogenesis as indicated by upregulated expression of α-SMA (8.3-fold) was further averted by Empa cotreatment which lessened the expression of α-SMA by 5.2-fold (Fig. 4).

### Examination of histological changes in the rats’ experimental groups using H & E stain

**Figure 5:**
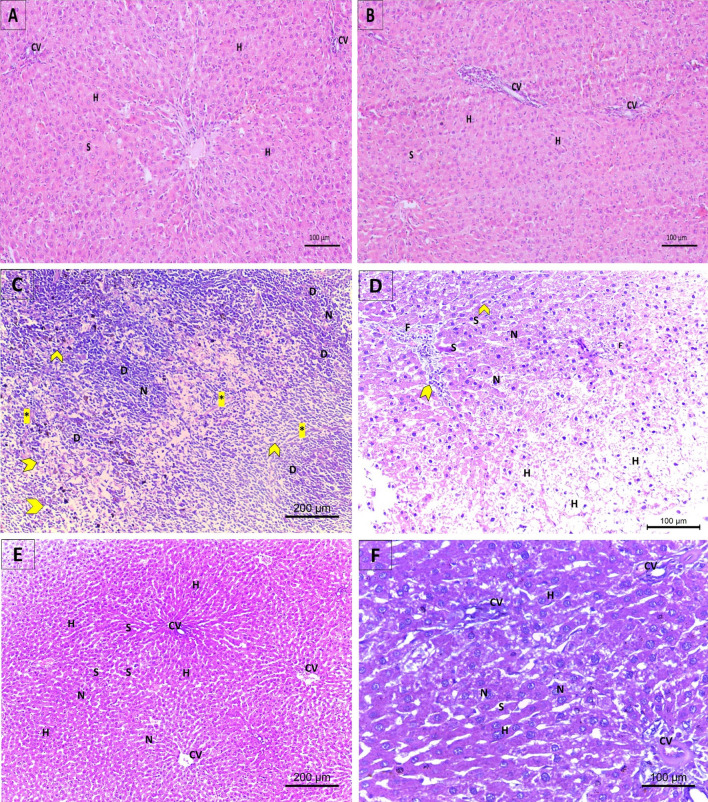
Examination of the effects of CCl_4_ and administration Empagliflozin in rats on the histopathology of the pericentral zone of hepatic tissues using H&E stain. (**A**), (**B**) Control and empagliflozin only groups show arrangement of hepatocytes (H) in cords around the central vein (CV) with vesicular nuclei and intact parenchyma and sinusoids (S). (**C**), (**D**) CCl_4_ treated group exhibits extensive degeneration of hepatic parenchyma (D), losing the polygonal hepatic cells buildup some of which are ballooned others have fallen out (H), foci of cellular necrosis (F), pyknotic nuclei (N), flood of mononuclear lymphocytes and other inflammatory cells (arrowhead), empty cytoplasm, dilation of sinusoids (S) and domination of collagen fibers (asterisk). (**E**, **F**) Cotreatment with empagliflozin in the CCl_4_ group resulted in regain of hepatic cell organization (H), restore of vesicular nuclei (N), departure of inflammatory cells and slightly dilated sinusoids (S).

**Figure 6:**
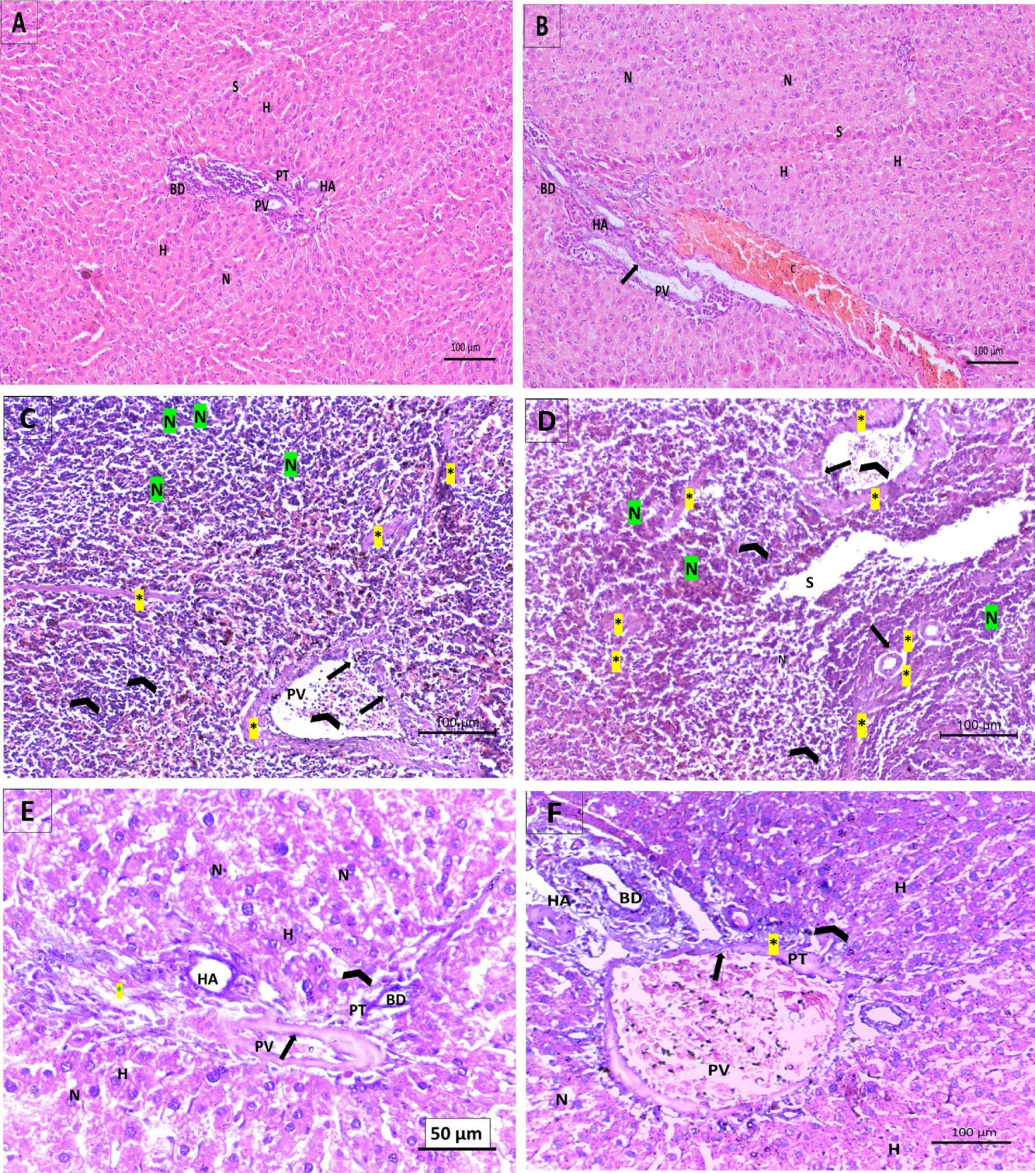
Examination of the effects of CCl_4_ and administration Empagliflozin in rats on the histopathology of the periportal zone of hepatic tissues using H&E stain. (**A**, **B**) Control and empagliflozin only groups show regular shaped hepatocytes (H) with prominent vesicular nuclei (N), tight peri-portal hepatic sinusoids (S), intact vasculature and draining system indicated by a sealed portal triad (PT) composed of portal vein (PV), bile duct (BD) and hepatic artery (HA). (**C**, **D**) CCl_4_ treated group displays remarkable cellular damage and nuclear pyknosis (N), extremely dilated sinusoids (S), deposition of collagen bundles (asterisk), interrupting the hepatic circulation and hardening the hepatic tissues. The portal vein (PV) has thickened fibrous wall (asterisk), which is also perforated (long arrow), piling the inflammatory cells (arrowhead). (**E**, **F**) After cotreatment with empagliflozin in the CCl_4_ group, there were hexagonal hepatic cells with active nuclei (N). Portal vein (PV), portal artery (HA) and bile duct (BD) have defined walls lined with endothelial cells. Occasionally, vascular congestion (C) was found around the portal triad. Collagen fibers (asterisk) and inflammatory cells (arrowhead) were within normal limit.

**Figure 7:**
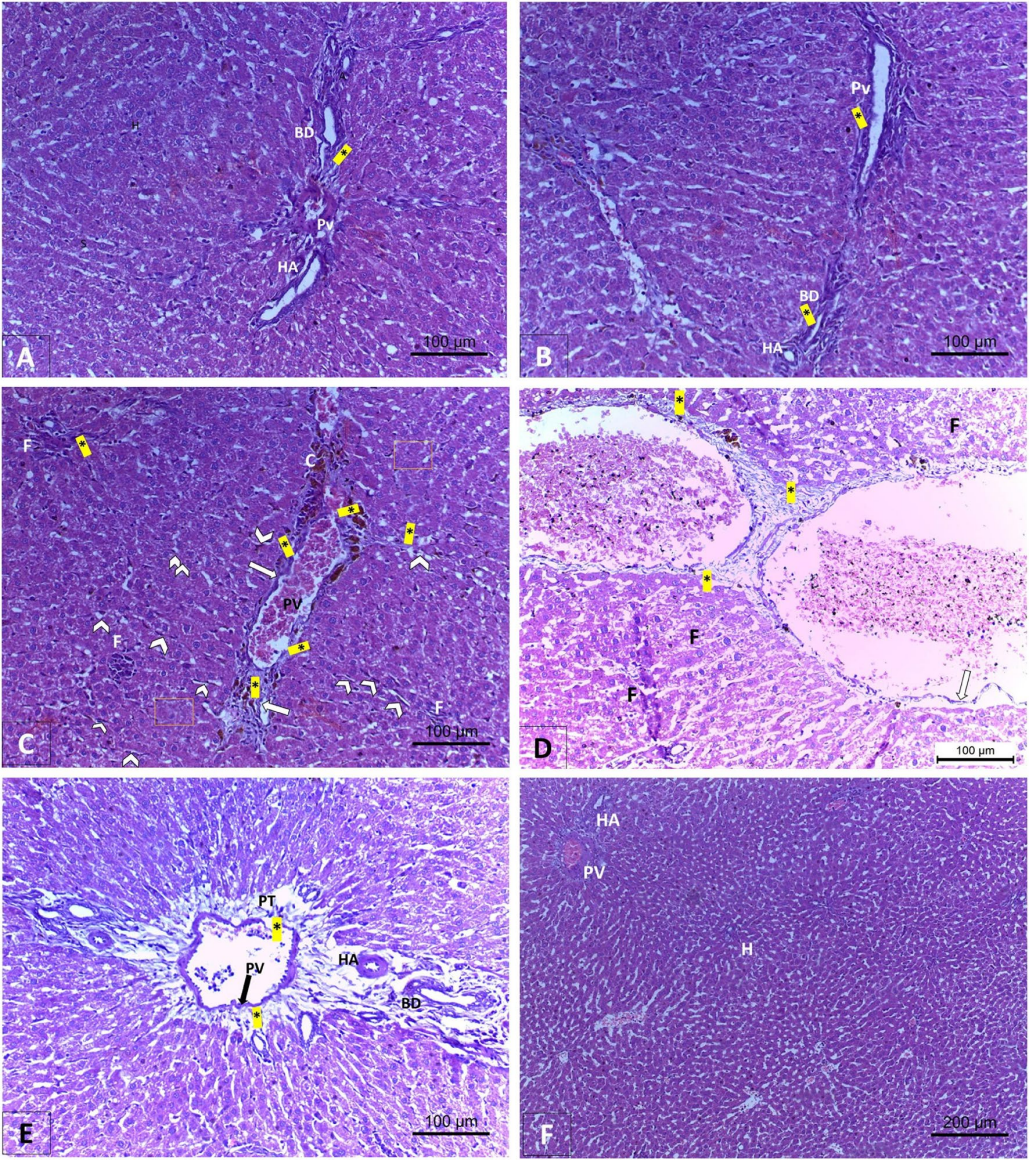
A Examination of the effects of CCl_4_ and administration Empagliflozin in rats on the histopathology of the periportal zone of hepatic tissues using Masson's trichrome stain. (**A**, **B**) In the control and empagliflozin only groups, there is appropriate amount of collagen fibers (asterisk) amidst the portal triad (portal vein (PV), bile duct (BD) and hepatic artery (HA), indicating proper inflow of blood and outflow of bile. (**C**, **D**) CCl_4_ treated group exhibits buildup of collagenous filaments (asterisk) and inflammatory cells (arrowhead), together with congested vessels. In another micrograph, the central vein (CV) is having collagen fibers around the walls group and with dislocated endothelial lining (long arrow). Focal areas of necrotic cells were widespread (**F**). (**E**, **F**) After cotreatment with empagliflozin in the CCl_4_ group, collagen deposits (asterisk) are diminished around the portal triad (PT) and central veins (CV) which led to regular hepatic architecture.

**Figure 8:**
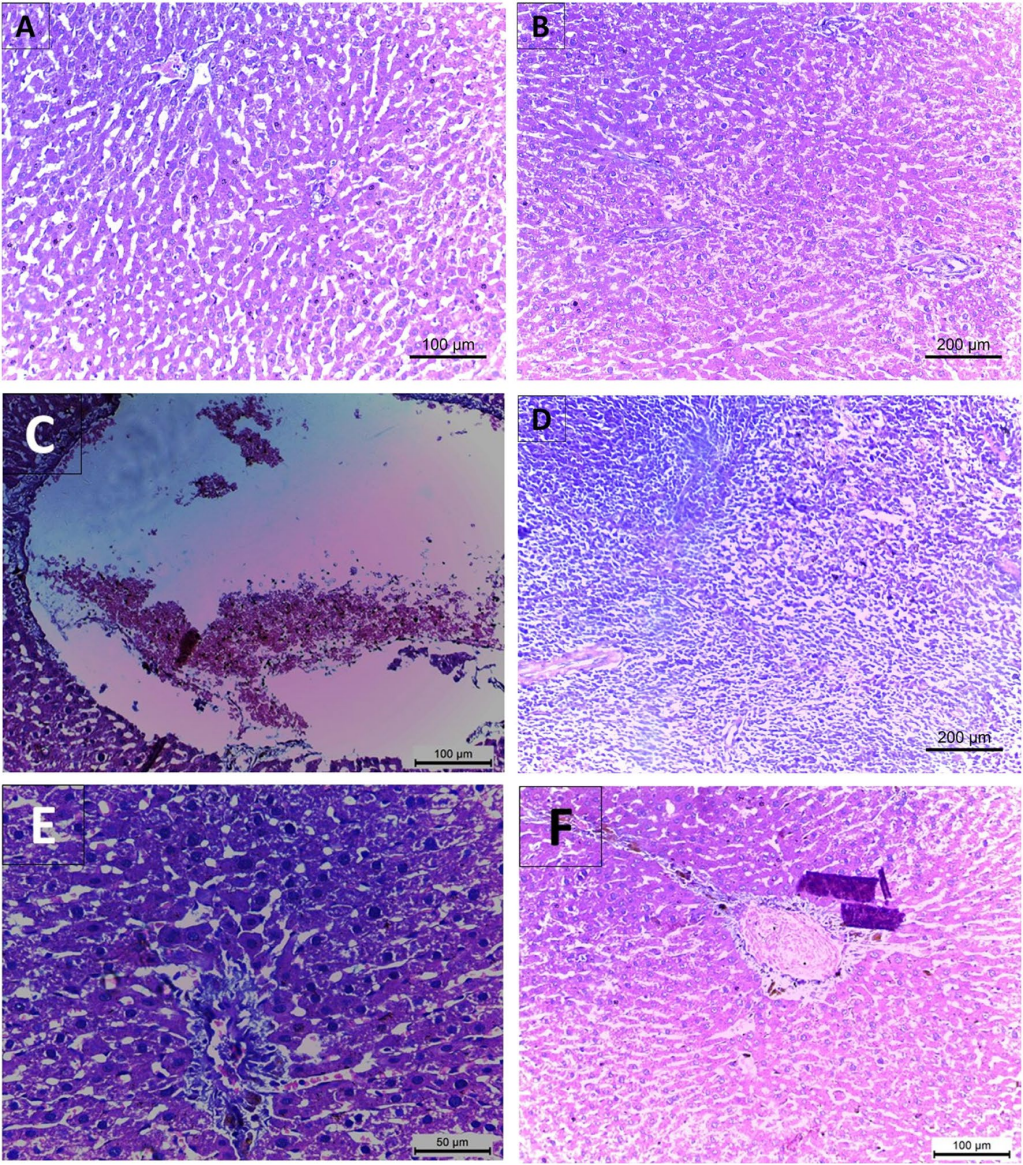
Ishak fibrosis score in rats after administration of CCl_4_ and cotreatment with Empagliflozin. (**A**, **B**) Stage 0 in the control and empagliflozin only groups, (**C**, **D**) CCl_4_ treated group exhibits stage 3. (**E**, **F**) Stage 1 Ishak score is shown by cotreatment with empagliflozin in the CCl_4_ group.

Examination of the histopathology of the pericentral zone of hepatic tissues is shown in Fig. 5. Control and Empa only groups displayed normal hepatic parenchyma organization consisting of hepatocytes organized in cords around the central vein with vesicular nuclei and surrounded with narrow sinusoids. CCl_4_ intoxication elicited extensive degeneration of hepatic parenchyma, recruitment of mononuclear lymphocytes and other inflammatory cells, dilation of sinusoids and domination of collagen fibers. Cotreatment with empagliflozin preserved hepatic cell organization, induced departure of inflammatory cells but sinusoids remained slightly dilated. Examination of the histopathology of the periportal zone of hepatic tissues is shown in Fig. 6. Control and Empa only groups showed intact vasculature and draining system as indicated by a sealed portal triad composed of portal vein, bile duct and hepatic artery. In the empagliflozin only groups, vascular congestion was occasionally found around the portal triad. In the CCl_4_ treated group, there was broad deposition of collagen bundles, interrupting the hepatic circulation and hardening the hepatic tissues. The portal vein had thickened, fibrous and perforated walls and harbored population of inflammatory cells. In the CCl_4_/Empa group, collagen fibers and inflammatory cells were within normal limit, which is proposed to help revitalize hepatocytes and restore hepatic construction. When Masson’s trichrome stain was applied to examine the histopathology of the periportal zone of hepatic tissues, the collagen fibers were clearly visible. In the control and empagliflozin only groups, the amount of collagen fibers was modest amidst the portal triad, indicating proper blood inflow and bile outflow. In the CCl_4_ treated group, the buildup of collagenous filaments which also filled the central vein was escorted by widespread areas of necrotic cells. In the CCl_4_/Empa group, collagen deposits were diminished around the portal triad along with regular hepatic architecture (Fig. 7). The qualitative assessment of fibrosis through Ishak scoring revealed that the control and Empa only groups had stage 0, while CCl_4_ group had significant liver fibrosis (Ishak score that ranged from 3 to 4). The endpoint for recovery was defined as histological improvement of fibrosis in form of a decrease of at least 1 Ishak score, in this regard the CCl_4_/Empa group exhibited amelioration of liver fibrosis (Ishak score = 1) (Fig. 8).

### Examination of Ultrastructure in the rats’ experimental groups using TEM

**Figure 9:**
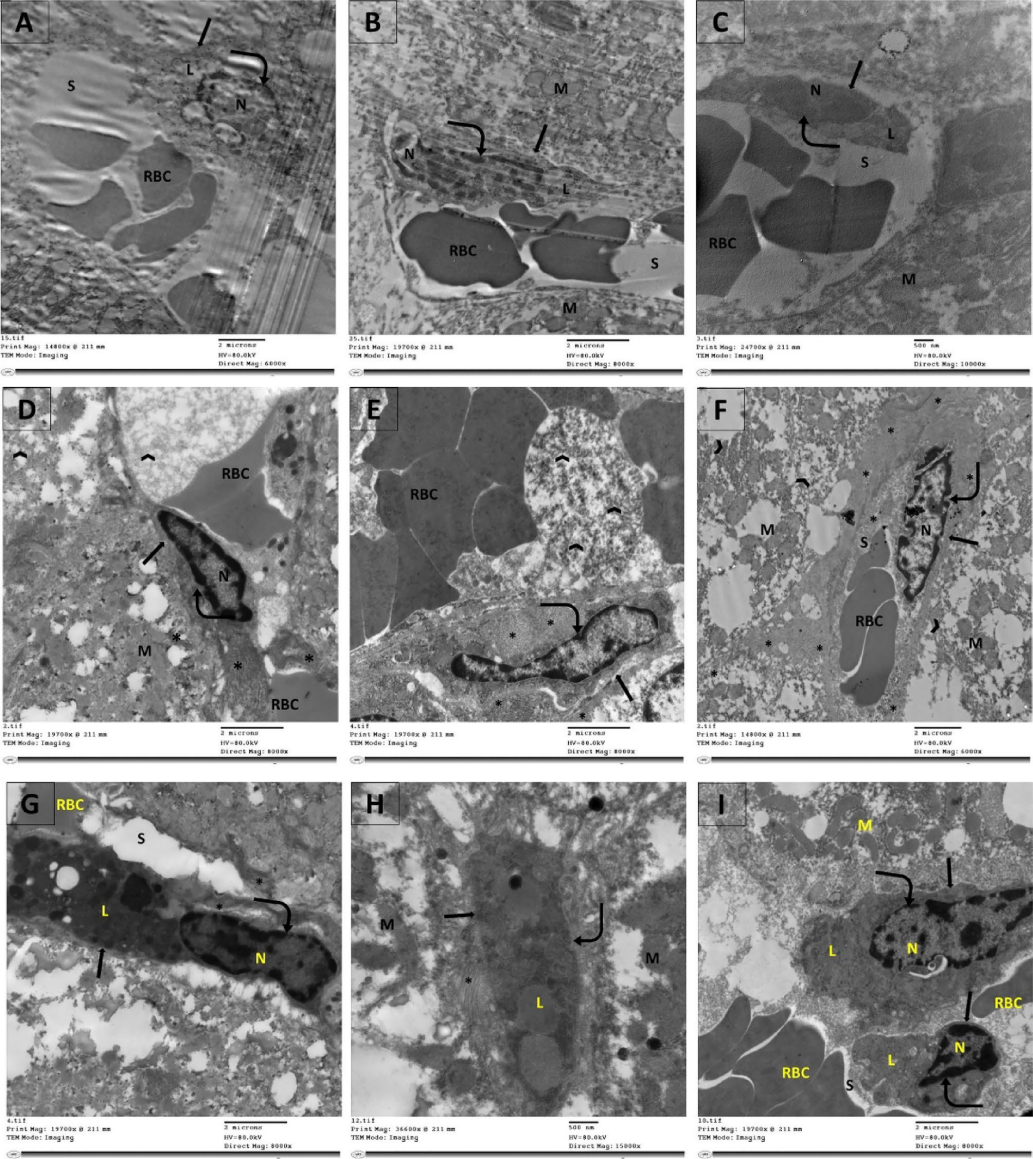
Examination of the effects of CCl_4_ and administration Empagliflozin on the periportal zone ultrastructure of hepatic tissues using TEM imaging technique. (**A**, **B**) Control group and (**C**) empagliflozin only groups show HSC (arrow) in the pericyte stage with nucleus (N) of smooth nuclear membrane (bent arrow) and vitamin A lipid droplets (L). The sinusoid (S) has normal blood flow (RBC). Hepatocytes (H) have normal mitochondria (M). (**D**–**F**) CCl_4_ treated group. In D and E, the myofibroblast (arrow), once it was HSC, is secreting fibrillar matrix (asterisk), filling its own cytoplasm and the surrounding cells. ER shreds are (arrowhead) reaching out to the adjacent sinusoid (S), obstructing it. In F, the myofibroblast is apoptotic (arrow) with shredded nucleus (N), ruffled nuclear membrane (bent arrow), fibrous deposits (asterisk), blocking the sinusoidal space (S)and the surrounding RBCs. Disruption of blood supply and dispersion of fibrous matrix within the hepatic parenchyma (asterisk) highly impact the hepatocytes which display irregular mitochondria (M) and worn ER (arrowhead), backfiring to induce apoptosis of myofibroblast. (**G**–**I**) After treatment with empagliflozin in the CCl_4_ group, the HSCs (arrow) show smooth nuclear membrane (bent arrow), structural lipid components (L) and normal amount of fibrous deposit (asterisk). Sinusoidal space (S) is clear from fibrous deposits and cell fragments.

**Figure 10:**
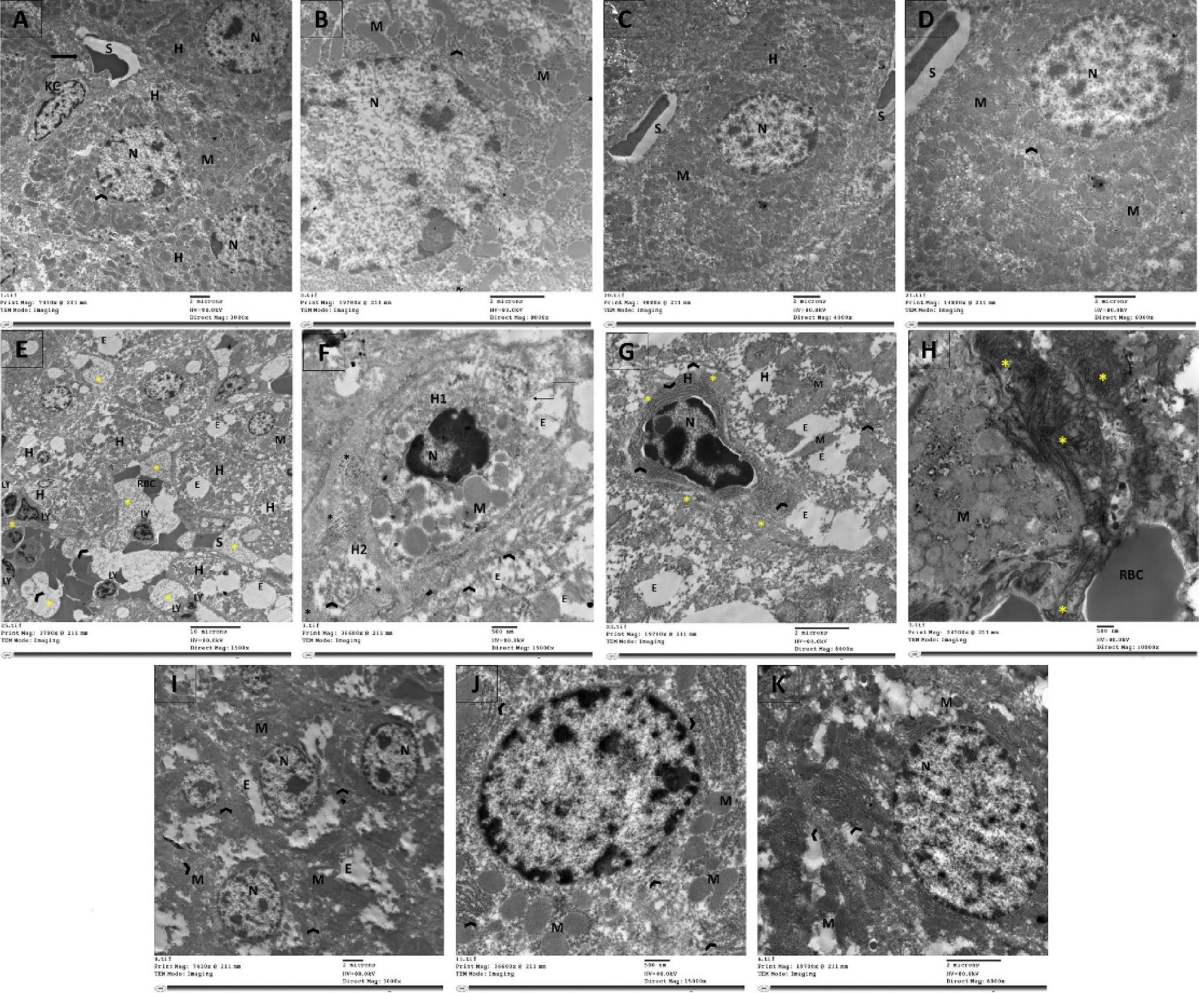
Examination of the effects of CCl_4_ and administration Empagliflozin on the pericentral zone ultrastructure of hepatic tissues using TEM imaging technique. (**A**, **B**) Control group and (**C**,** D**) empagliflozin only groups, they all show hepatic parenchymal cells consist of definite polygonal hepatocytes (H) with rounded nuclei (N), numerous elongated mitochondria (M) and normal ER (arrowhead). The blood sinusoids (S) are clear and surrounded by a dormant HSC (arrow) and Kupfer cell (KC). B and D represent higher magnification of A and C and show intact nucleus (N) surrounded by cytoplasm packed with elongated and rounded mitochondria (M) and disperse ER (arrowhead). (**E**–**H**) CCl_4_ treated group. In E, apoptotic hepatocyte (H) are widespread in the hepatic parenchyma with pyknotic shrunken nuclei (N), wide empty cytoplasmic spaces (E). Sinusoidal vessels (S) are clogged with ER fragments (arrowhead) and fibrous secretion (*). Inflammatory cells such as lymphocytes (LY) are jamming the hepatic milieu. F represents a dying hepatocyte (H1), having nucleus with marginated chromatin (N), swollen mitochondria (M) of different size and low in number, broken cell membrane (elbow arrow), fibrillar aggregates (*) and ER cut into small pieces (arrowhead). In a waning hepatocyte (H2), the nucleus has been broken down and massive aggregate of fibers (*) encircle twirl of ER (arrowhead). In G, fibrous filaments (*) enclose apoptotic hepatocyte (H) in which the nucleus (N) is pyknotic, the mitochondria (M) are absent, and the ER (arrowhead) is dilated. The adjacent hepatocytes have fused or distorted mitochondria, empty cytoplasm, and fragmented ER (arrowhead). In H, Huge fibers are invading the hepatic milieu which displays fused mitochondria. (**I**–**K**) The animal group treated with empagliflozin after CCl_4_ exhibits regular shape hepatic cells with active nuclei, abundant mitochondria within balanced ER network and smaller cytoplasmic empty spaces (E).

Examination of the ultrastructure of the periportal zone of hepatic tissues is shown in Fig. 9. Control and empagliflozin only groups had normal flowing sinusoid lined with pericyte-stage HSCs saturated with vitamin A lipid droplets. In the CCl_4_ treated group, the HSCs have metamorphosed into myofibroblasts which secrete fibrillar matrix. The sinusoidal space is blocked by ER shreds and collagen fibers. A general state of cellular demise of hepatocytes and sometimes myofibroblasts is observed. In the CCl_4_/Empa group, the HSC phenotype, few fibrous deposits, and clear sinusoidal space were detected. Examination of the ultrastructure of the pericentral zone of hepatic tissues is shown in Fig. 10. In the control group and empagliflozin only groups, the hepatic parenchymal cells consisted of definite polygonal hepatocytes and the blood sinusoids were clear and surrounded by a dormant HSC. In CCl_4_ treated group, apoptotic hepatocytes were prevalent, and the sinusoidal vessels were clogged with ER fragments and fibrous secretion. Inflammatory cells such as lymphocytes were frequent in the hepatic milieu. In the CCl_4_/Empa group, hepatic cells adapted hexagonal shape with active nuclei, abundant mitochondria within balanced ER network and smaller cytoplasmic empty spaces.

## Discussion

Given the insidious onset, asymptomatic course, and one million annual deaths worldwide, the fathom of liver fibrosis remains far from accomplished. In fact, the significant regenerative capacity of the liver makes the development of fibrosis in a damaged liver unexpected^31^. From another perspective, increased resilience in hepatic tissues is the culprit in fibrosis, as it promotes expeditious replacement of damaged cells with connective tissues, while the solution is the creation of new cells^32^ (Supplementary Fig. 1).

CCl_4_ model of hepatic fibrosis translates the trajectory of liver fibrosis in human as it implements liver strategic detoxification of xenobiotics, for which hepatocytes are equipped with plethora of heme containing enzymes called cytochrome P450 (CYP) fibrosis^2^. Of which, CYP2E1 catalyzes hydroxylation of hydrogen containing lipophilic toxins to their hydroxylated soluble derivatives^33^. In case of CCl_4_ which has no hydrogen, it is reduced by CYP2E1 to trichloromethyl free radicals (˙CCl_3_), an obnoxious oxidizing agent that undergo oxidative damage of the hepatic ultrastructure and macromolecules^34^.

In this study, CCl_4_ treated rats had elevated MDA levels and depleted GSH and SOD content, consistent with previous studies reporting CCl_4-_ triggered disruption of membrane lipids and subcellular damage^35^. Hence, hepatic lesion developed in response to CCl_4_, provided a context of activated immune response and induced proinflammatory cytokines^36^. In our study, increased hepatic TNF-α and IL-6 after CCl_4_ administration indicate the abundance of M1 macrophages^37^. Profuse TNF-α and IL-6 reciprocate by making M1 macrophages the predominant phenotype^37^. In this study, remarked elevation of IL-17, a proinflammatory cytokine, refers to the differentiation of naïve T cells to Th17 cells which induce robust inflammatory response and participates in HSCs activation^38^. In our study, TGF-β, a pleiotropic ligand, exhibited over expression in response to CCl_4_ application. Earlier, TGF-β has shown spike expression in oxidative environment to collaborate with IL-6 to skew the naïve T helper cells to become Th17^39^. Additionally, we found inactive expression of IL-10; a cytokine secreted by Treg in immunosuppressive environment^40^.

Even though inflammation is a reaction of the immune system aiming at healing injured tissues, exacerbated release of inflammatory cytokines turns into attacking hosting tissues, spreading damage^11^.

As the ER is the intracellular site where CCl_4_ detoxification takes place, ER suffers from the proceedings of this toxicant^41^. In this study, downregulation of ERAD expression in CCl_4 -_treated rats declares failure of cytoprotection by UPR response^13^. On the other hand, high expression levels of CHOP denote commencement of apoptosis of hepatocytes^14^.

In the current study, CCl_4_- induced hepatocytes injury was further evident by elevated ALT and AST serum levels and impaired synthetic functions was detected by decreased albumin levels in serum. Injured cells produce damage- associated molecules that can bind and activate Hh receptors in HSCs, promoting their survival and transition to collagen secreting myofibroblasts^1^. Active expressions of Ptch1, Smo and Gli-2 were detected in our study after intoxication with CCl_4_. Previously, aberrant Hh signaling was found to target HSCs, making them more viable, also liable to switching into myofibroblasts^42^. In our study, the myofibroblasts marker; α-SMA, was highly expressed in CCl_4_- rats compared to the control rats, evidencing the profusion of myofibroblasts^43^. Histologically, we found aggregates of collagen fibers and ER shreds clogging the sinusoidal vessels and portal triad, disrupting the blood supply to the parenchymal cells. Also, ECM was dissipated within the pericentral zone of hepatic tissues, to further sabotage the cellular communication and hepatic hemodynamic. Inflammatory cells have infiltrated within the hepatic vessels and parenchyma.

In view of the serious impact of the liver fibrosis on the globe, it became a priority to exploit conventional drugs to reverse the fibrosis or at least to keep the status quo. A precondition for selecting a suitable conventional drug is the ability to intervene early in the pattern of hepatic fibrosis. In the present study, administration of Emba in CCl_4_ rats encumbered oxidative stress as demonstrated by the increase in the hepatic levels of SOD and GSH and the decrease in MDA levels. The current findings are aligned with the Empa putative role in activation of the nuclear factor erythroid 2 (Nrf2)- related factor 2 pathway^44^. Our results showed that coadministration of Emba in CCl_4_ rats resulted in pronounced anti-inflammatory effect as displayed by decreased hepatic levels of TNF-α and IL-6, downregulated expression of IL-17 and upregulated IL-10 expression. These findings are in line with recently published studies which proved the anti-inflammatory actions of SGLT-2 inhibitors through activation of Toll-like receptor 4/nuclear factor-kappa B signaling pathway^45^. In this study, we found overexpression of IL-10 after Empa cotreatment, verifying shifting of M1 macrophages to M2 macrophages which have scavenging properties and are capable of eradicating residual damage^46^. Previously, SGLT-2 inhibitors were implicated in polarizing M1 macrophages to M2 macrophages and promoting immunosuppression through Treg^47^. Specifically, Empa improved fat utilization through polarization of M2 macrophages in high fat diet mice^48^. In this study, Empa/CCl_4_ rats had elevated levels of TGF-β that were insignificantly different from the CCl_4_ rats. Evidently, TGF-β acts as immunomodulator and may stimulate or suppress the immune response according to the setting and the redox state^49^. Therefore, we suggest that TGF-β acted as a proinflammatory cytokine in accordance with CCl_4_ and as anti-inflammatory growth factor after coadministration of Empa.

In our study, the placated hepatic tissue did not show active expression of CHOP, while ERAD was overexpressed, which portrays the success of the mechanism of protein quality control in response to Empa cotreatment. This overall recuperative mode is suggestive of diminished damage related molecules and their putative roles as ligands for Hh pathway. In this regard, we found that the Hh pathway was inhibited in the Empa/CCl_4_ rats, as Ptch1, Smo and Gli-2 were not actively expressed. Recently, a few reports investigated the association between ER stress and activation of Hh pathway. Conforming with our result, one report found that suppressing Hh pathway inhibited CHOP and ER stress thereby mitigated pulmonary fibrosis^50^. Another report, however, indicated that activating Hh pathway was linked to improvement of ER stress and hepatic function in diabetic model^51^.

In the current study, Empa cotreatment inhibited α-SMA expression in hepatic tissues relative to the CCl_4_ group. These findings provide evidence that Empa inhibits HSCs activation and implicates the Hh pathway as a prerequisite for the transformation of HSCs into myofibroblasts. The histological and cellular images supported the biochemical findings as we found that in the Empa/ CCl_4_ rats, HSCs displayed structural lipid components, characterizing quiescent HSCs. Also, sinusoidal space was clear from fibrous deposits and cell fragments, this aspect was reflected on the tenacity of hepatocyte architecture.

Our results showed that cotreatment with EMPA significantly reduced hepatocytes damage and increased hepatocytes efficiency as indicated by decreased serum levels of hepatic enzymes, ALT and AST, and increased albumin serum levels. As an SGLT2 inhibitor which does not stimulate insulin secretion, EMPA has the advantage of not increasing the risk of hypoglycemia^52^.

Future studies to explore the full panel of fibrosis markers and Hh pathway through Western blotting are recommended. It is also warranted to visualize the fibrosis markers by immunostaining and confirm the collagen formation by more stains such as Sirius Red.

## Conclusion

We confirmed the anti-fibrotic effect of EMPA in CCl_4-_ intoxicated rat model. After cotreatment with EMPA, the net decrease in fibrogenic cells abundance and increase in hepatic cells survival are apparently ascribed to the antioxidant and anti-inflammatory actions of Empa. By further elaboration we found that the hepatoprotection of Emba is partly attributed to inhibiting signaling of Hh pathway which is supposed to arrest the HSCs in their innate forms, the vitamin A loaded cells. We also propose an association between Hh pathway and ER stress where Empa-induced inactive Hh signaling increases quality control of misfolded protein disposal and decreases ER stress-driven apoptosis.

### Supplementary Information

Supplementary Figure 1.

Supplementary Figure 2.

Supplementary Figure 3.

Supplementary Figure 4.

## Data Availability

The data that support the finding of this study are available from the corresponding author upon reasonable request.
