# Supplementary figures and images for: Empagliflozin suppresses hedgehog pathway, alleviates ER stress, and ameliorates hepatic fibrosis in rats
Source: Sci Rep. 2023 Nov 3;13:19046. doi: 10.1038/s41598-023-46288-5 (PMC10624673; doi:10.1038/s41598-023-46288-5)

## Supplementary Figure 1

### Amplification Plot of ERAD

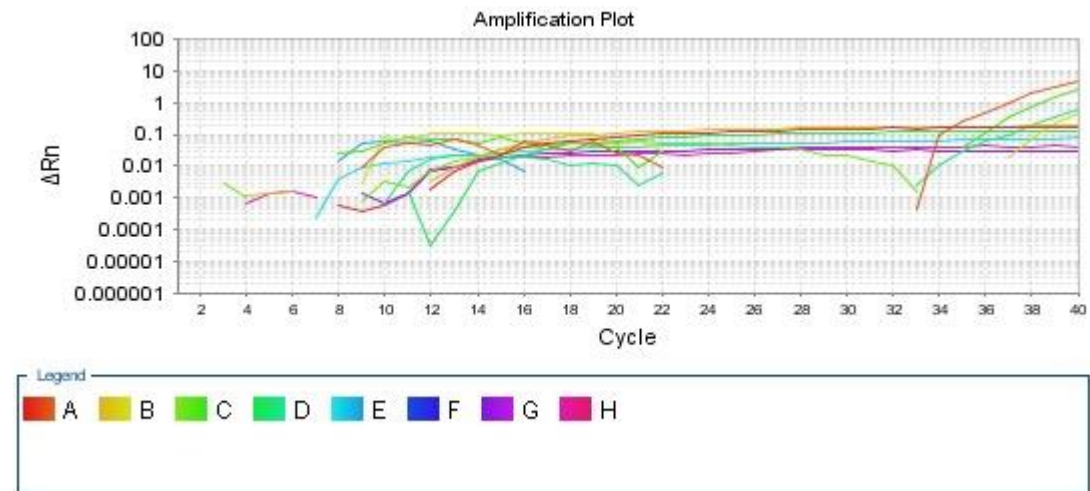

### Amplification Plot of CHOP

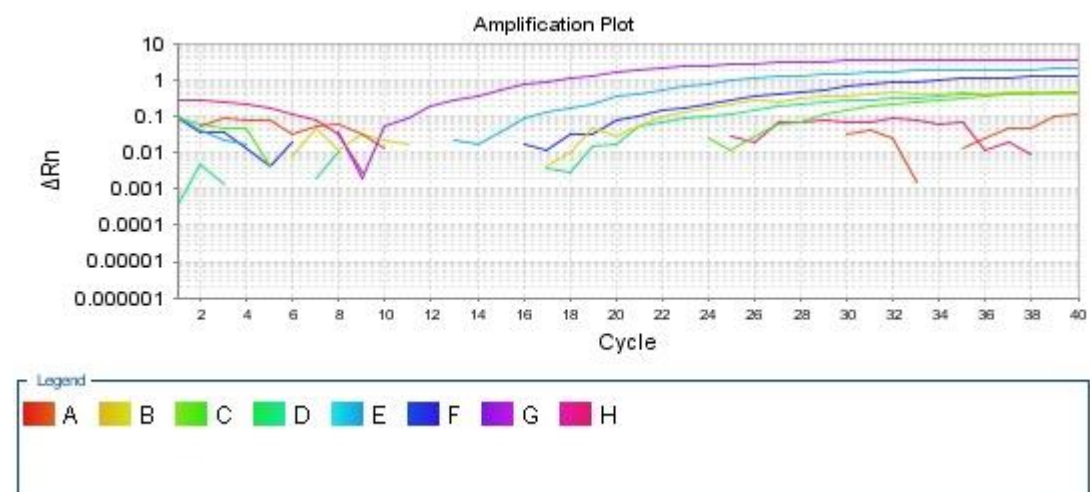

Supplement: Supplementary file 1 — Supplementary Figure 1. [file 41598_2023_46288_MOESM1_ESM.pdf]

Supplementary Figure 2

Amplification Plot of IL-10

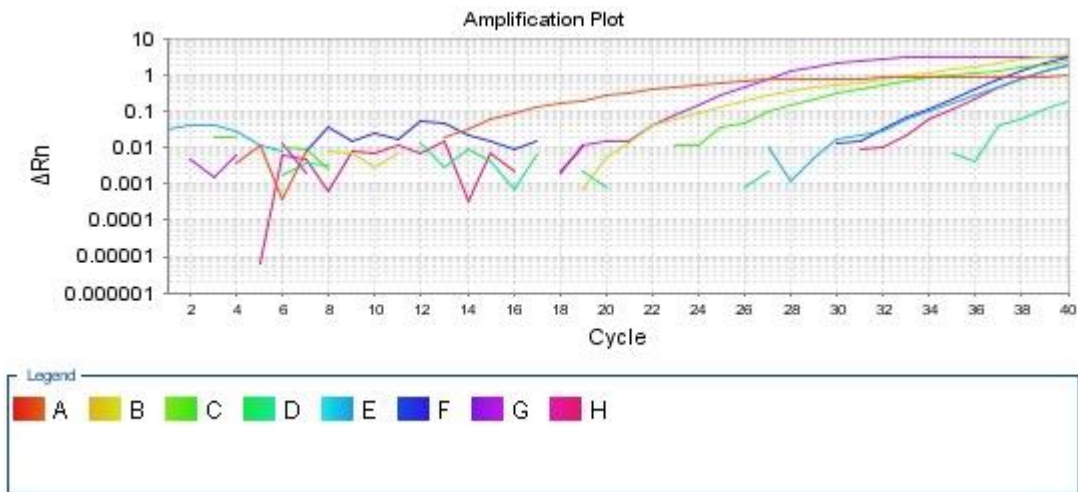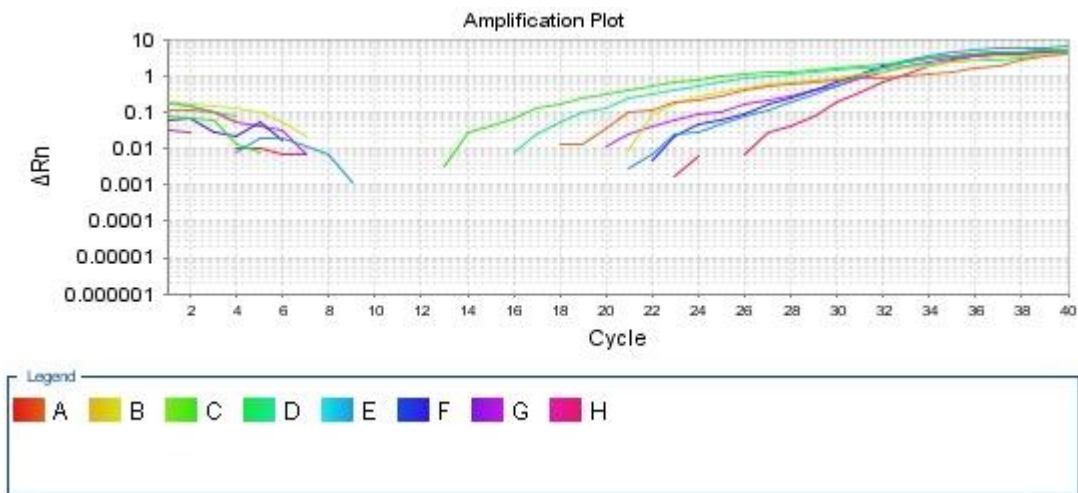

Supplement: Supplementary file 2 — Supplementary Figure 2. [file 41598_2023_46288_MOESM2_ESM.pdf]

### Supplementary Figure 3

#### Amplification Plot of IL-17

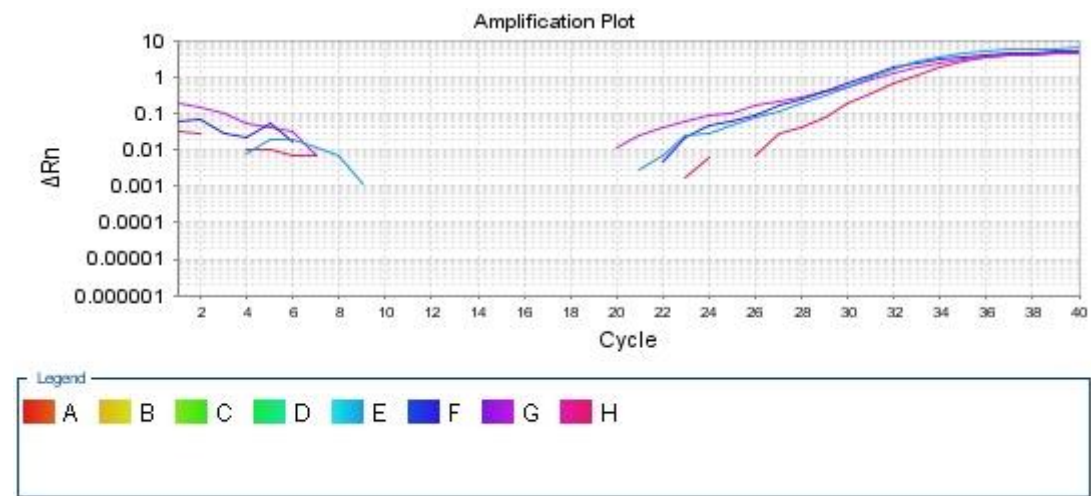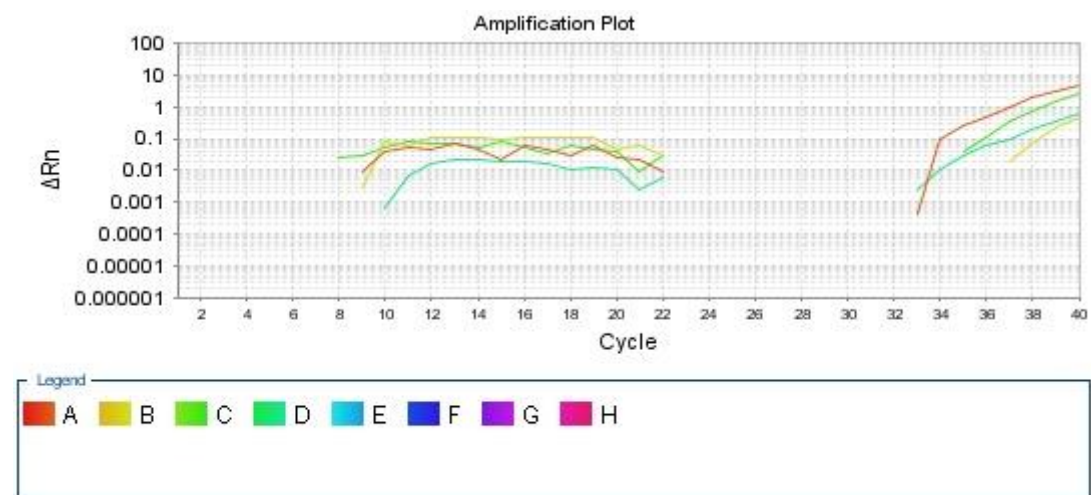

Supplement: Supplementary file 3 — Supplementary Figure 3. [file 41598_2023_46288_MOESM3_ESM.pdf]
