# Supplementary material for: Empagliflozin suppresses hedgehog pathway, alleviates ER stress, and ameliorates hepatic fibrosis in rats
Source: Sci Rep. 2023 Nov 3;13:19046. doi: 10.1038/s41598-023-46288-5 (PMC10624673; doi:10.1038/s41598-023-46288-5)

Supplementary Figure 4

Amplification Plot of Ptch-1

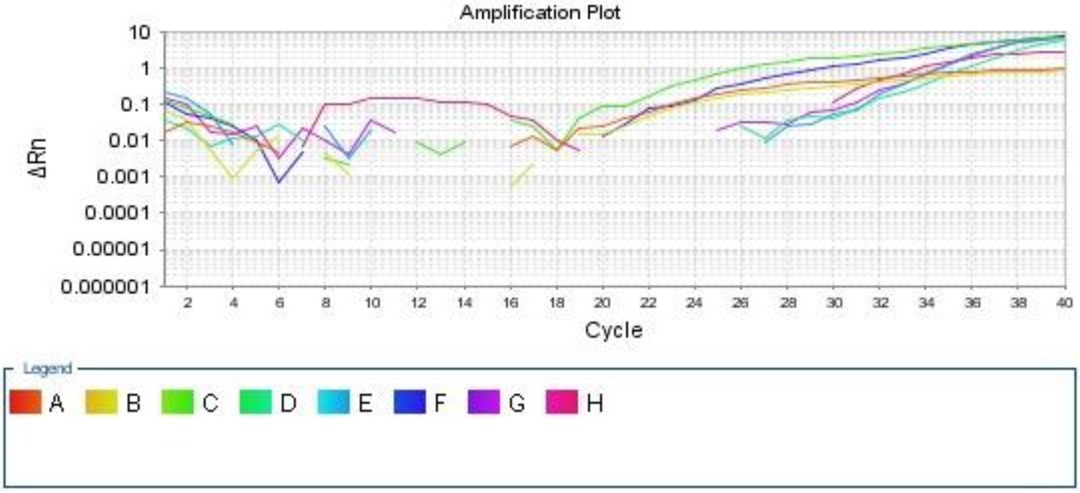

Amplification Plot of Smo

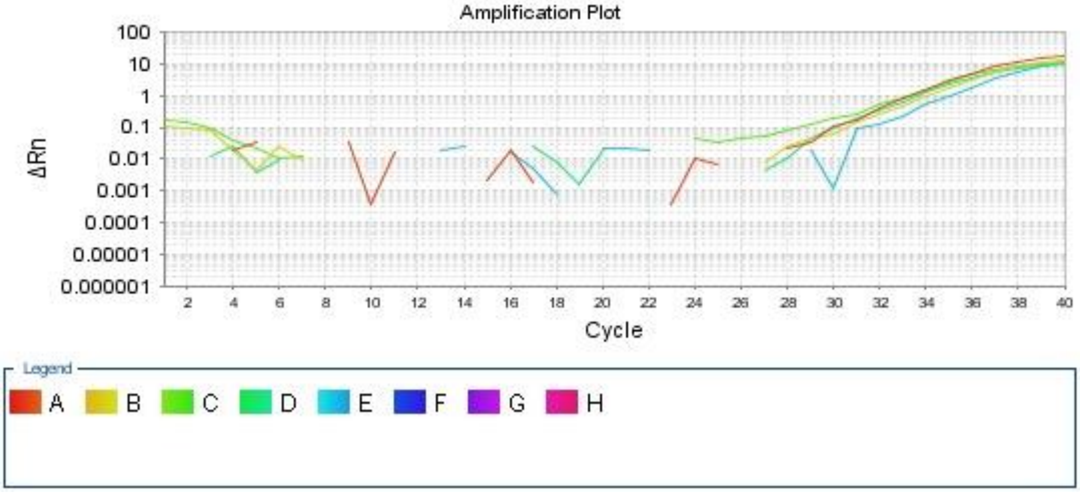

Supplementary Figure 4

Amplification Plot of Gli-2

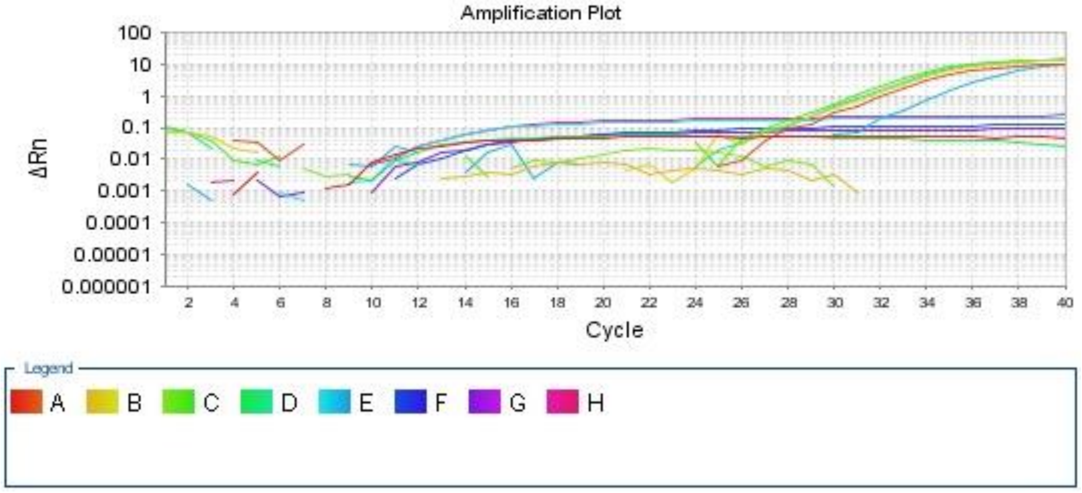

Amplification Plot of α-SMA

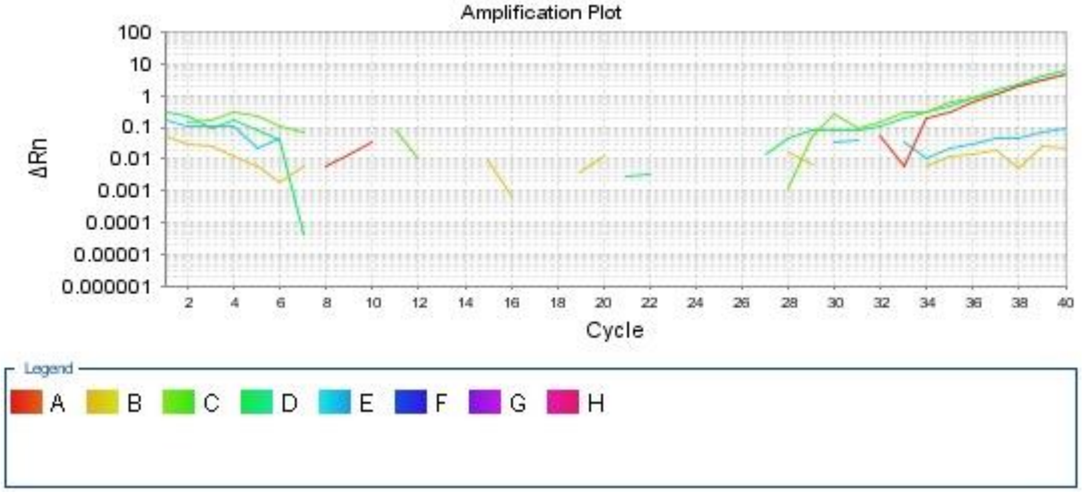

Supplement: Supplementary file 4 — Supplementary Figure 4. [file 41598_2023_46288_MOESM4_ESM.pdf]
